# Frequency of diseases and use of medicinal plants in adults in a rural Peruvian community

**DOI:** 10.3389/fpubh.2026.1806021

**Published:** 2026-05-07

**Authors:** Claudia Mercedes Reyna Gonzales, Sonia Celedonia Huyhua-Gutiérrez, Omer Cruz Caro, Rosa Jeuna Diaz-Manchay, Sonia Tejada-Muñoz

**Affiliations:** 1 Escuela Profesional de Enfermería, Universidad Nacional Toribio Rodríguez de Mendoza de Amazonas, Chachapoyas, Peru; 2 Facultad de Ciencias de la Salud, Escuela Profesional de Enfermería, Instituto de Investigación en Salud Integral Intercultural, Universidad Nacional Toribio Rodríguez de Mendoza de Amazonas, Chachapoyas, Peru; 3 Oficina de Gestión de la Calidad, Universidad Nacional Toribio Rodríguez de Mendoza de Amazonas, Chachapoyas, Peru; 4 Escuela Profesional de Enfermería, Universidad Católica Santo Toribio de Mogrovejo, Chiclayo, Peru

**Keywords:** epidemiological profile, primary health care, self-care practices in health, sociocultural determinants, traditional medicine

## Abstract

In rural communities, the use of medicinal plants remains a widely practiced traditional health strategy closely linked to local morbidity patterns and sociocultural factors. The aim of this study was to describe the distribution of self-reported illnesses and to characterize the use of medicinal plants according to disease type, recurrence, and selected sociodemographic characteristics among adults in a rural community in Luya, Amazonas, Peru. A descriptive–analytical cross-sectional study was conducted with 400 adults using validated structured questionnaires administered during household visits between January and February 2025. Illness episodes were defined as any health condition reported during the 3 months preceding the survey and were classified according to the International Classification of Diseases (ICD-10). In total, 14 reported disease items and 18 symptom items were identified and grouped into 13 ICD-10 disease categories. Descriptive statistics, chi-square tests, and multivariable ordinal logistic regression were applied. The most frequent disease category corresponded to respiratory diseases (32.5%), followed by musculoskeletal (19.8%) and digestive conditions (13.5%). Medicinal plant use was common, with infusions (34.8%) and decoctions (12.2%) as the main preparation methods. Chi-square analysis identified significant associations between medicinal plant use and sex, educational level, illness duration, healthcare utilization, symptoms, and perceived usefulness (*p* < 0.05). However, in the multivariable model, only age (OR = 1.022; 95% CI: 1.011–1.034; *p* < 0.001) and sex (OR = 0.550; 95% CI: 0.383–0.789; *p* = 0.001) remained significant predictors. These findings indicate that medicinal plant use is primarily structured by sociodemographic factors rather than by direct indicators of disease burden. The results provide relevant evidence on self-care practices in rural contexts, highlighting the sociocultural importance of medicinal plants in health-seeking behavior.

## 1 Introduction

The use of plants with medicinal properties and traditional medicine methods is a healthcare strategy deeply rooted in many rural communities globally, especially in situations where access to conventional medical services is expensive, scarce, or culturally distant (1–3). In addition to cultural and social factors, reliance on traditional medicine is also shaped by structural barriers, including limited financial protection, high out-of-pocket expenditures, and restricted access to formal health services in rural areas (4). These conditions influence health-seeking behavior, leading households to adopt home-based remedies as initial or alternative care strategies (5). In this context, traditional medicine plays a complementary role to biomedical care by offering accessible and socially accepted alternatives perceived as effective for managing common symptoms, especially in rural contexts and vulnerable populations (6, 7).

The World Health Organization (WHO) acknowledges that traditional, complementary, and integrative medicine systems, which include the use of plants with medicinal properties, are used in more than 170 countries. It is estimated that between 40% and 99% of populations use some form of traditional medicine in their daily healthcare (8). These practices remain an affordable, accessible, and culturally acceptable alternative, especially in rural and remote areas. Furthermore, they are part of self-care and symptom management decisions for several common illnesses (8).

The frequency and type of diseases present in a population directly influence health care seeking strategies (9, 10). In rural communities, people alternate or combine home remedies, healers, and biomedical services depending on the type and severity of the illness (11, 12). Studies in Brazil, Rwanda, India, and Pakistan show that plants are used primarily for frequent and mild symptoms, while more serious problems are referred to biomedical services, creating a practical complementarity in daily care (13–16).

Medicinal plants are considered a traditional source for the treatment of various ailments (6). Thus, their use is a common practice in rural communities in Asia (15, 17), Africa (18–21), and Latin America (22–24). According to epidemiological evidence, plants are the foundation of primary health care and coexist with formal biomedical systems. In Peru and other Andean nations, ethnobotanical research indicates that a large part of the population uses plant-based remedies to treat urinary, respiratory, and digestive disorders (25–27), gastrointestinal disorders, and chronic conditions (28–30). This reflects both the region's biological diversity and the intergenerational transmission of traditional knowledge.

Ethnobotanical studies, which document traditional knowledge of the use of medicinal plants, are essential for the discovery of new drugs and for understanding the relationship between biodiversity and cultural practices (31, 32). Despite the continued expansion of the pharmaceutical industry, the world still relies on ethnomedicine to treat basic illnesses (33), particularly in rural communities where traditional medicine remains widely used (34–37). This continued reliance is largely explained by the accessibility, affordability, and cultural embeddedness of medicinal plants within local health-care practices, especially when compared with more costly pharmaceutical alternatives (38). However, it is important to emphasize that the traditional or ethnomedicinal use of plants does not necessarily guarantee safety or therapeutic efficacy (39, 40). Therefore, the scientific validation of these resources is essential and requires comprehensive approaches, including phytochemical characterization, pharmacological analyses, and biological evaluation of active compounds, which contribute to understanding their mechanisms of action and establishing their therapeutic potential (41–43). In this sense, integrating traditional knowledge with rigorous scientific approaches is crucial to promote the informed and safe use of medicinal plant resources (44).

Knowledge of medicinal plants remains central to community health, but it is unevenly distributed within populations; recent empirical and synthesis studies show consistent intracultural differences linked to age, gender, education, livelihoods, and domestic roles (45–47). Beyond compiling species inventories, recent research has increasingly emphasized the importance of understanding how traditional medicinal plant knowledge varies among social groups within communities and how these differences shape local health practices and knowledge transmission (46, 48, 49).

Despite the development of ethnobotanical studies in the Peruvian Amazon, there remains a limited integrated description of the co-occurrence patterns between medicinal plant use and reported illnesses from epidemiological and sociodemographic perspectives, particularly in rural communities (50, 51). The literature has largely focused on the taxonomic description of species or on specific therapeutic applications, leaving aside the descriptive analysis of recurrence, reported frequency, and the functional impact of illnesses on the daily lives of the population. This gap is particularly relevant in the Peruvian Amazon, where health problems linked to intestinal parasitosis, diarrheal diseases, and respiratory infections persist, often aggravated by conditions of social and nutritional vulnerability (52, 53).

In addition, rural settings are frequently characterized by unequal access to health services, geographical barriers, long distances to care facilities, and limited local health infrastructure, which together shape health-seeking behavior and patterns of service use. These conditions also contribute to small-area and geographic variations in healthcare utilization and reporting patterns, which may not necessarily reflect true differences in underlying morbidity but rather place-based disparities in access, behavior, and resource constraints (54). In territorially dispersed contexts, spatial accessibility becomes a key determinant of self-care practices and may lead to the substitution or complementarity of formal health services with traditional medicine (5, 55). In this context, which is also characterized by persistent limitations in access to essential medicines, the use of medicinal plants constitutes a culturally embedded self-care strategy supported by the region's high biological diversity.

Within this framework, it is relevant to describe the reported illnesses and patterns of medicinal plant use in specific rural populations, as this allows for a better understanding of self-care practices, perceived health needs, and their relationship with sociodemographic characteristics. In addition, health-related decision-making in rural contexts often occurs within households and communities through participatory and socially negotiated processes, where women and older adults frequently play a central role in treatment selection and knowledge transmission (56). Considering these dynamics is essential to better interpret behavioral responses such as the use of medicinal plants and to explain potential differences associated with sex and educational level. In this context, the present study focuses on adults from a rural community in northern Peru with the aim of describing the distribution of self-reported illnesses and the use of medicinal plants, as well as their distribution according to disease type, recurrence, and selected sociodemographic characteristics among adults in a rural community in Luya, Amazonas, Peru. By integrating social and cultural variables, this research seeks to provide descriptive evidence on co-occurrence patterns and health self-care practices in rural contexts.

## 2 Materials and methods

### 2.1 Design

The study employed a quantitative, non-experimental, cross-sectional, descriptive–analytical design. This design allowed for the characterization of the distribution of self-reported illnesses and medicinal plant use among adult participants from a rural community, as well as the exploration of associations between these variables and selected sociodemographic characteristics.

### 2.2 Study population

**Figure 1:**
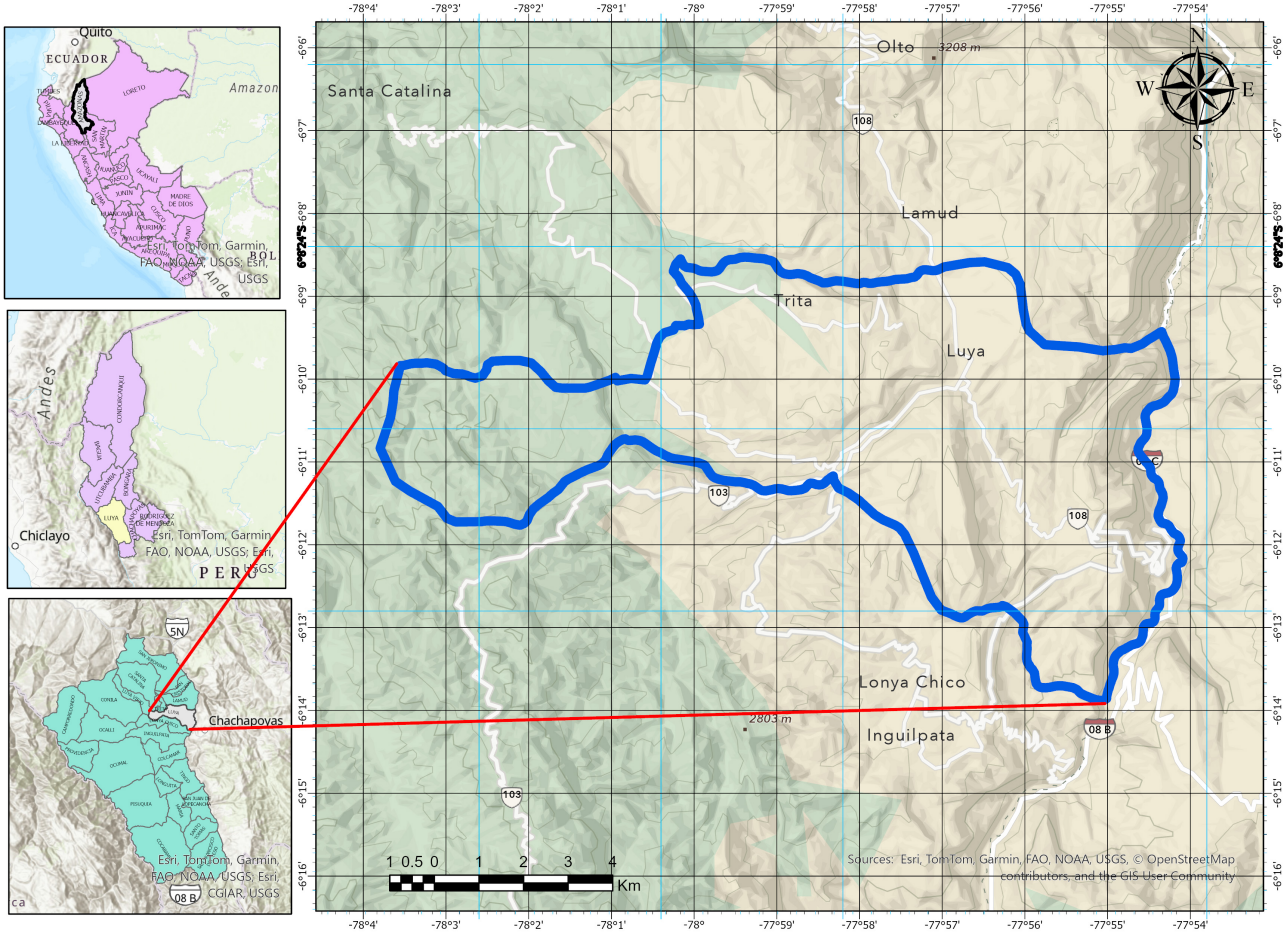
Location of the study area.

The Luya District (Figure 1) is located in northeastern Peru, in the Amazonas region (6°09′53^′′^ S, 77°56′40^′′^ W). The average altitude within the study area is 2,300 m above sea level, with a total population of 4,118 inhabitants and a population density of 43.41 inhabitants/km^2^, reflecting its predominantly rural and dispersed character. It is a predominantly rural community with low population density and settlements distributed between the district capital and various hamlets, where the local economy is based primarily on small-scale agricultural activities.

The district's geographical and ecological conditions, typical of the Andean-Amazonian region, are associated with a high availability of plant resources, which has supported the persistence and intergenerational transmission of traditional health practices. However, it should be noted that the ecological availability and distribution of medicinal plant species within the district were not directly measured in this study and are inferred from regional ecological characteristics. In addition, the district of Luya has a single primary health care facility, located in the city of Luya, which serves as the main point of access to formal health services for the surrounding rural population.

The present study focused on the reported use of medicinal plants as part of self-care practices and did not include a taxonomic or ethnobotanical characterization of the species used. Therefore, no botanical identification, voucher specimen collection, or standardized plant collection protocols were performed. The information on medicinal plant use was based on self-reported data provided by participants, reflecting local knowledge and practices rather than formally validated botanical records. Future research should incorporate ethnobotanical identification procedures and phytochemical profiling of frequently cited species to enable a more comprehensive understanding of their pharmacological properties and to support the transition from traditional use to scientifically validated evidence (57).

The information was collected using family record forms administered during household visits conducted by the health promotion team of the local health center, composed of nursing professionals, as part of community health promotion and prevention activities. During these visits, households located in the four neighborhoods of the district were approached, and adult residents who were present at the time of the visit agreed to participate voluntarily were invited to complete the questionnaire after providing informed consent. Therefore, the sample corresponds to a non-probabilistic community sample based on voluntary participation during household visits.

This recruitment strategy may introduce selection bias related to participant availability and willingness to participate, as only individuals present at the time of the visit were included. However, the use of household visits across multiple neighborhoods allowed broad community coverage and facilitated the inclusion of participants from diverse local contexts within the district.

Non-probabilistic sampling refers to a technique in which participants are selected based on accessibility and availability rather than through random selection (58). This approach was considered appropriate given the rural and geographically dispersed nature of the study population, as well as the reliance on household visits conducted by local health personnel, which facilitated access to participants available at the time of data collection.

For analytical purposes, the records were organized according to the district's territorial distribution into four neighborhoods, as presented in Table 1.

**Table 1:** Distribution of respondents by sector.

Sector	Frequency	Percentage
Labrador	77	19.3
San Jose	117	29.3
Santa Cruz	93	23.3
Guadalupe	113	28.1
**Total**	**400**	**100.0**

A total of 400 participants were included in the study, corresponding to the eligible individuals identified during household visits across the four neighborhoods, which allowed broad coverage of the study population and ensured sufficient variability for the descriptive analysis.

The study included individuals over 18 years of age residing in the study area who voluntarily agreed to participate after signing an informed consent form. The overall sample comprised adult participants identified during household visits. For analytical purposes, a subsample was defined including only participants who reported having experienced an illness and whose condition could be classified according to the International Classification of Diseases, tenth revision (ICD-10, 2019 version). Consequently, the analysis describes the distribution of reported conditions within this subsample and does not allow inference of disease frequency at the population level.

### 2.3 Information gathering

Information was collected between January and February 2025 through the administration of structured questionnaires to participants who met the established inclusion criteria. To assess the distribution of self-reported illnesses, a questionnaire specifically designed for this study was used, consisting of eight questions organized into three sections: (i) frequency and duration of illness episodes during the previous 3 months, (ii) diagnosis and symptoms reported by the participants, and (iii) the impact of illnesses on daily activities. These questions included categorical response options related to the frequency of disease occurrence, number of visits to health care facilities, duration of illness episodes, associated symptoms, and functional limitations.

For the variable medicinal plant use, a structured questionnaire composed of three questions was employed to assess the frequency of medicinal plant consumption, the perceived usefulness of medicinal plants for the treatment of illnesses, and the forms of preparation or use (e.g., decoction, infusion, extract, compress, or inhalation). The first two questions used ordinal frequency scales with five response categories (ranging from “never” to “always”), while the third allowed respondents to select multiple forms of use. However, the instrument was designed to capture general patterns of use and perceived usefulness, and did not include detailed information on species identification, dosage, treatment duration, or safety-related aspects. Therefore, findings on medicinal plant use should be interpreted within this descriptive scope. Future studies should incorporate pharmacological and toxicological assessments to better evaluate safety and potential risks (59).

The content validity of the instruments was assessed through expert judgment, in which specialists evaluated the clarity, relevance, and coherence of the items in relation to the study objectives. A pilot test was subsequently conducted with 30 participants with characteristics similar to those of the study population to assess clarity and contextual appropriateness. Given the multidimensional nature of the morbidity instrument, which includes frequency, duration, diagnosis, symptoms, and functional impact, internal consistency measures were not considered appropriate for all sections. Therefore, Cronbach's alpha was estimated only for the items related to medicinal plant use, yielding a value of 0.83, indicating adequate internal consistency for this specific scale.

### 2.4 Ethical aspects

The study was conducted in accordance with the ethical principles outlined in the Declaration of Helsinki (60). The data collection procedures were carried out in coordination with the local health center. All participants were informed about the objectives of the study and participated voluntarily after providing written informed consent. The information collected was treated confidentially and used exclusively for research purposes. The questionnaires were self-administered during household visits conducted by the local health center's health promotion team, ensuring privacy and standardized conditions for all participants.

### 2.5 Data analysis

Data analysis was performed using descriptive and analytical statistical procedures. Prior to analysis, self-reported diagnoses and symptoms were standardized by translating lay terminology into clinically equivalent terms and subsequently classified according to ICD-10 using predefined criteria. Each reported condition was coded independently, and ambiguous or non-specific responses were grouped into a residual “other” category. The coding process was reviewed by a researcher with training in health sciences to ensure consistency.

Descriptive statistics included absolute frequencies and percentages for sociodemographic variables, disease characteristics (type, frequency, duration), use of health services, and medicinal plant consumption. For variables allowing multiple responses, such as diseases and symptoms, percentages were calculated based on the total number of participants, acknowledging that categories are not mutually exclusive. Additionally, patterns of medicinal plant use according to disease profiles and sociodemographic characteristics were explored using percentage distributions and heat maps.

Bivariate associations between medicinal plant use and independent variables were assessed using chi-square tests, with statistical significance set at *p* < 0.05. Variables showing statistical significance or theoretical relevance were subsequently included in the multivariable analysis.

Finally, a multivariable ordinal logistic regression model was fitted to evaluate the association between medicinal plant use and selected sociodemographic and health-related variables, controlling for potential confounding factors. Results are presented as odds ratios (OR) with 95% confidence intervals (CI). Model fit was assessed using Akaike Information Criterion (AIC) and Bayesian Information Criterion (BIC), prioritizing parsimony and interpretability in model selection.

## 3 Results

**Figure 2:**
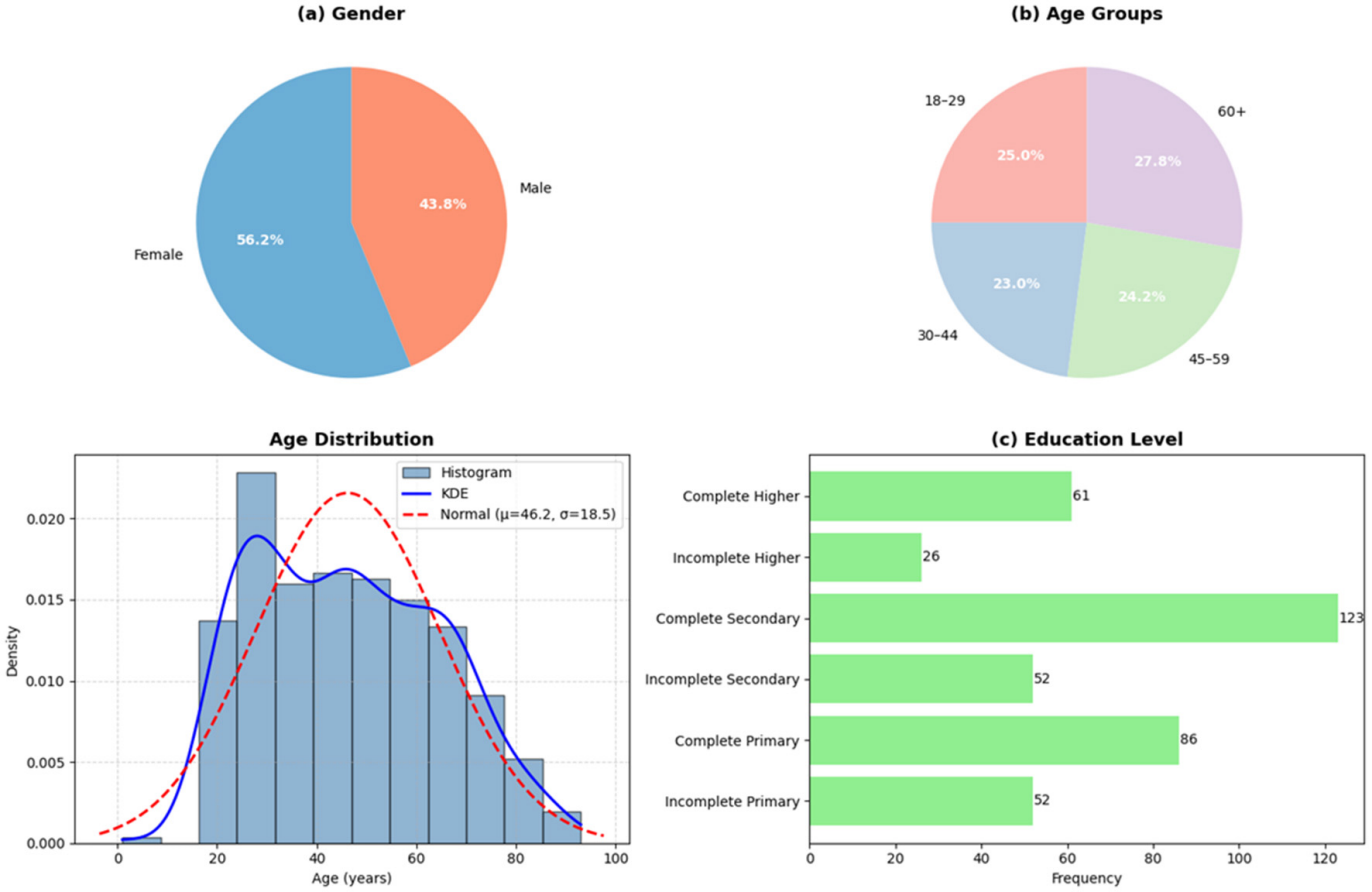
Sociodemographic characteristics of the study population. **(a)** Percentage distribution by sex. **(b)** Percentage distribution by age groups. **(c)** Distribution of the age of the participants. **(d)** Distribution of the educational level attained.

Figure 2 summarizes the sociodemographic characteristics of the 400 participants included in the study. Women accounted for 56.2% (*n* = 225) of the sample, while men represented 43.8% (*n* = 175). The distribution across age groups was relatively balanced, with 25.0% (*n* = 100) aged 18–29 years, 23.0% (*n* = 92) aged 30–44 years, 24.2% (*n* = 97) aged 45–59 years, and 27.8% (*n* = 111) aged 60 years or older. Regarding educational level, complete secondary education was the most frequent category (*n* = 123), followed by complete primary education (*n* = 86), whereas incomplete higher education (*n* = 26) and complete higher education (*n* = 61) were less frequent. These results are presented to describe the composition of the study sample and to provide context for subsequent analyses of health conditions and medicinal plant use.

**Figure 3:**
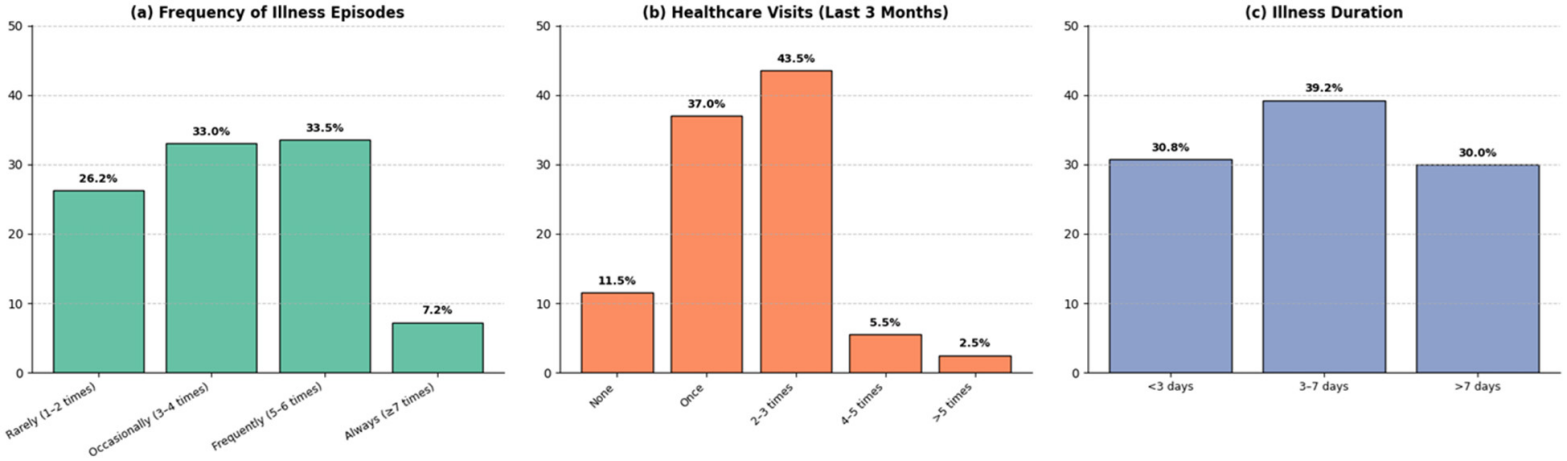
Indicators of disease frequency and use of health services in the last three months. **(a)** Frequency of reported diseases. **(b)** Number of visits to health centers due to disease. **(c)** Average duration of disease episodes.

Figure 3 presents indicators related to self-reported illness episodes, healthcare visits, and illness duration during the previous 3 months. Regarding illness frequency, 26.2% of participants reported experiencing health-related events rarely (1–2 times), 33.0% occasionally (3–4 times), 33.5% frequently (5–6 times), and 7.2% very frequently (≥7 times) during the reference period. It is important to note that these episodes correspond to self-perceived health events, which may include symptoms, discomfort, or recurrent conditions, rather than clinically distinct or medically confirmed illnesses.

Concerning healthcare utilization, 43.5% of participants reported visiting a health facility two to three times in the previous 3 months, followed by 37.0% who reported a single visit, whereas smaller proportions reported no visits or more than four visits. With respect to illness duration, 30.8% of reported episodes lasted < 3 days, 39.2% between 3 and 7 days, and 30.0% more than 7 days. These results describe perceived patterns of health events and healthcare utilization among participants during the reference period.

**Figure 4:**
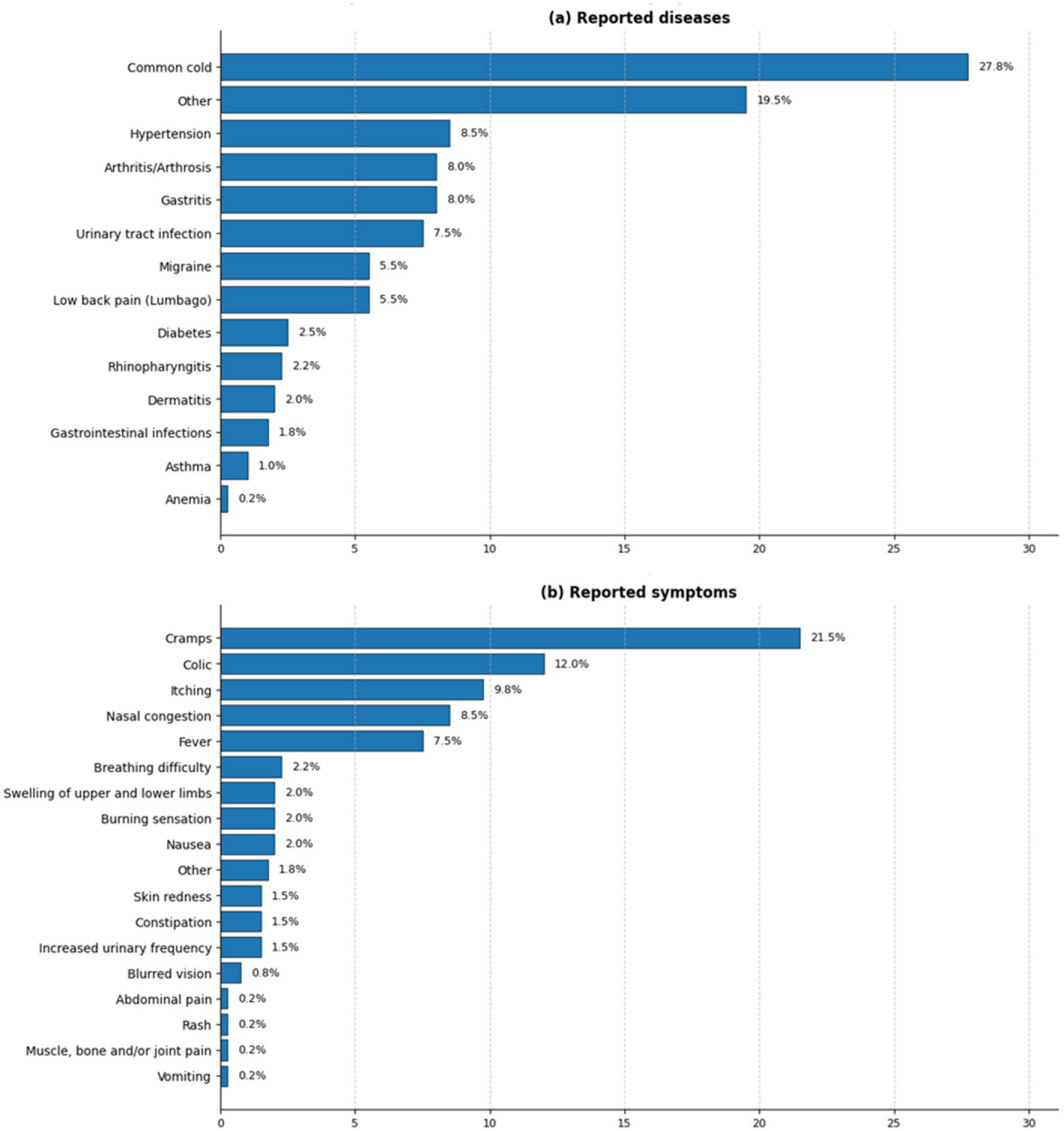
Diseases and symptoms reported by the study population. **(a)** Distribution of diseases reported by participants. **(b)** Distribution of symptoms associated with the reported diseases.

Figure 4 presents the self-reported illnesses and symptoms described by participants during the reference period, in total, participants reported 14 disease items and 18 symptom items. Among the most frequently reported conditions were common cold (27.8%, *n* = 111), followed by conditions grouped under the residual category “other” (19.5%, *n* = 78), hypertension (8.5%, *n* = 34), arthritis or arthrosis (8.0%, *n* = 32), gastritis (8.0%, *n* = 32), and urinary tract infection (7.5%, *n* = 30). Regarding self-reported symptoms, the most frequent were cramps (21.5%, *n* = 86), colic (12.0%, *n* = 48), itching (9.8%, *n* = 39), nasal congestion (8.5%, *n* = 34), and fever (7.5%, *n* = 30). These findings describe the distribution of self-reported health conditions and symptoms among participants and provide contextual information for the analysis of medicinal plant use. Reported conditions should be interpreted as participant-reported health events rather than clinically confirmed diagnoses.

**Figure 5:**
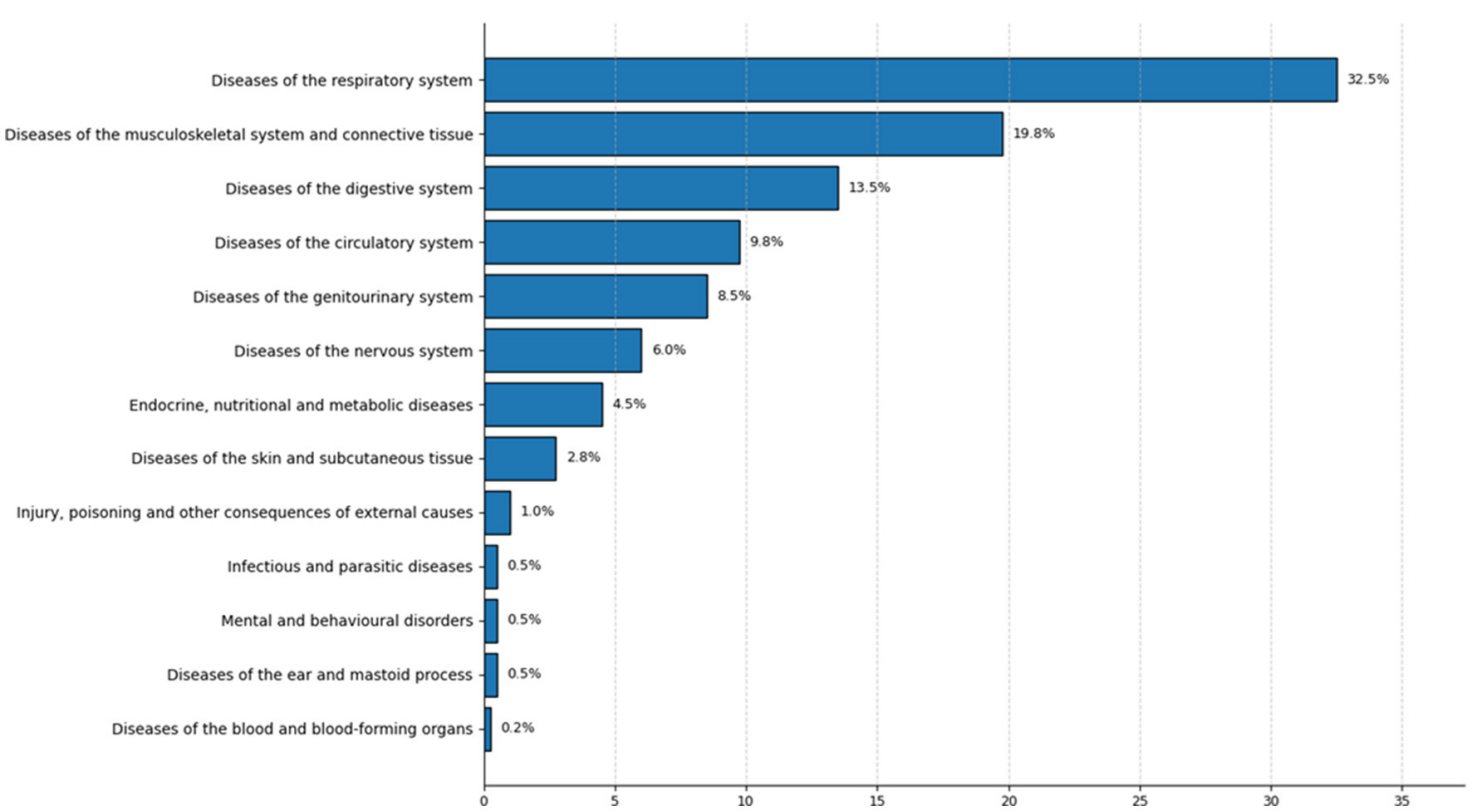
Distribution of reported diseases according to the International Classification of Diseases (ICD-10). The figure includes only the disease categories with the highest relative frequency according to the ICD-10 classification.

Figure 5 presents the distribution of self-reported illnesses classified according to ICD-10 chapters; the 14 reported disease items were grouped into 13 ICD-10 disease categories. The most frequent categories correspond to diseases of the respiratory system (32.5%), followed by diseases of the musculoskeletal system and connective tissue (19.8%) and diseases of the digestive system (13.5%). Lower proportions were observed for diseases of the circulatory system (9.8%), genitourinary system (8.5%), and nervous system (6.0%), while the remaining categories accounted for < 5% of reports.

It is important to note that these categories represent broad ICD-10 groupings, which may include heterogeneous conditions with different clinical characteristics (e.g., acute and chronic conditions within the same category). Therefore, the results should be interpreted as general patterns of self-reported morbidity rather than clinically specific disease profiles.

Because the study included only participants who reported experiencing an illness during the reference period, these findings reflect the distribution of reported conditions within the study sample and not population-level morbidity estimates. Categories with low frequencies should be interpreted with caution due to the limited number of cases.

**Figure 6:**
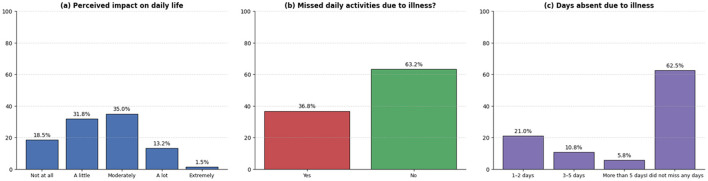
Impact of illnesses on daily life and absenteeism due to illness. **(a)** Perceived impact of illness on the ability to perform daily activities. **(b)** Absence from daily activities (work or study) due to illness. **(c)** Number of days of absence due to illness.

Figure 6 presents participants' perceptions of the impact of illness on their daily activities during the reference period. A total of 35.0% reported that illness moderately affected their ability to perform daily activities, while 31.8% indicated a slight impact and 13.2% reported a considerable impact. In contrast, 18.5% reported no impact and 1.5% reported an extreme impact.

Regarding the interruption of daily activities, 36.8% of participants reported having missed their usual activities due to illness, whereas 63.2% indicated that they had not. Among those reporting absences, the most frequent duration corresponded to 1–2 days (21.0%), followed by 3–5 days (10.8%) and more than 5 days (5.8%). These results describe the perceived functional impact associated with the illness episodes reported by participants during the reference period.

**Figure 7:**
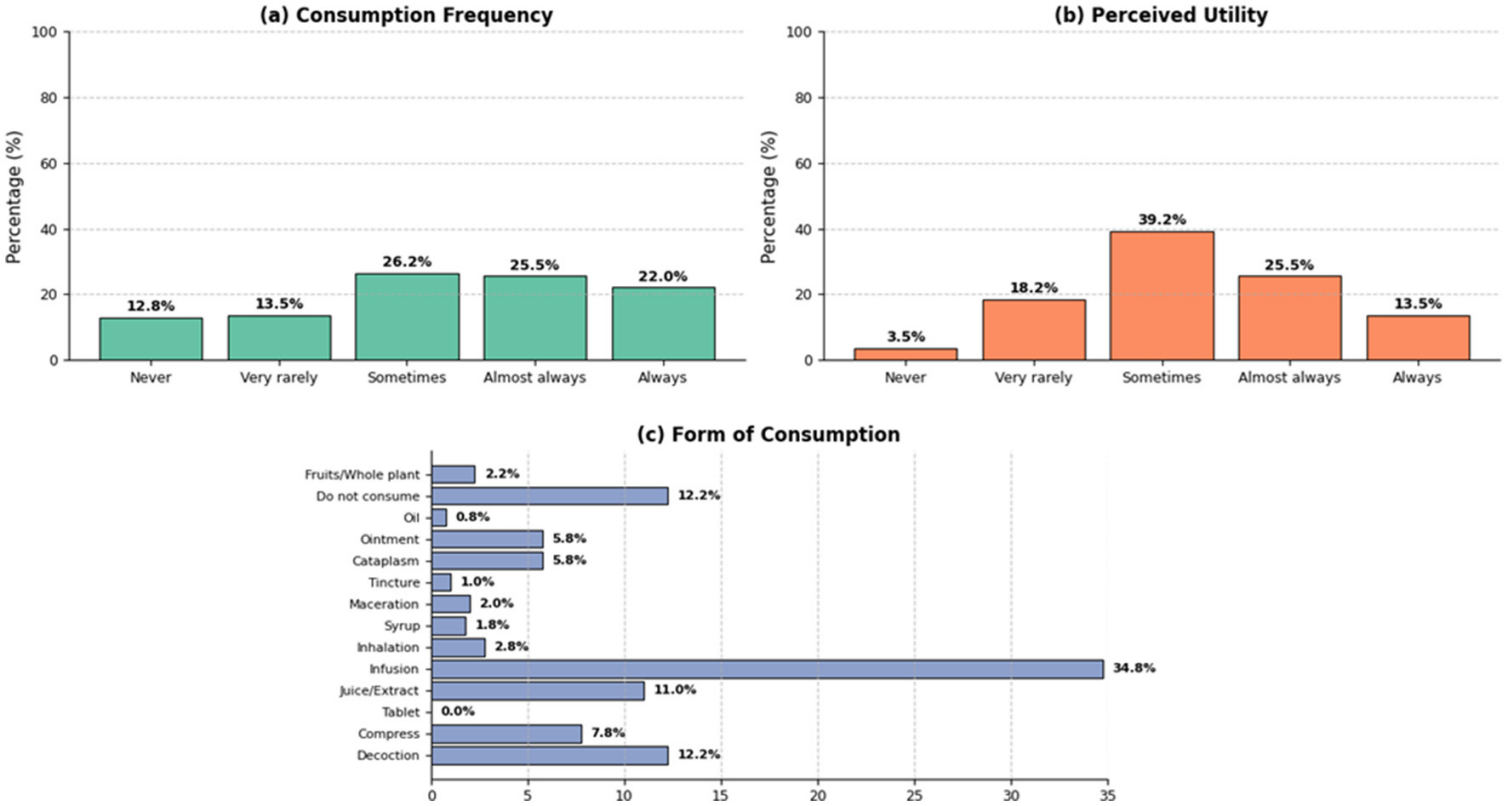
Frequency of consumption, perceived usefulness, and ways of using medicinal plants. **(a)** Frequency of consumption of medicinal plants. **(b)** Perceived usefulness of medicinal plants for the treatment of diseases. **(c)** Reported forms of consumption of medicinal plants.

Figure 7 presents the self-reported patterns of medicinal plant use among participants. Regarding frequency of consumption, a considerable proportion of the population reported using medicinal plants occasionally or frequently, whereas a smaller proportion indicated that they never or rarely use them. In terms of perceived usefulness, most participants reported that medicinal plants are useful or moderately useful for managing health-related conditions.

Concerning the forms of consumption, liquid preparations were the most frequently reported, particularly infusions (34.8%) and decoctions (12.2%), followed by juices or extracts (11.0%) and compresses (7.8%). Other preparation methods, such as poultices, macerations, ointments, or inhalations, were reported less frequently. These findings describe general patterns of use and preparation based on participant self-report and should not be interpreted as reflecting standardized therapeutic practices or specific pharmacological effects.

**Figure 8:**
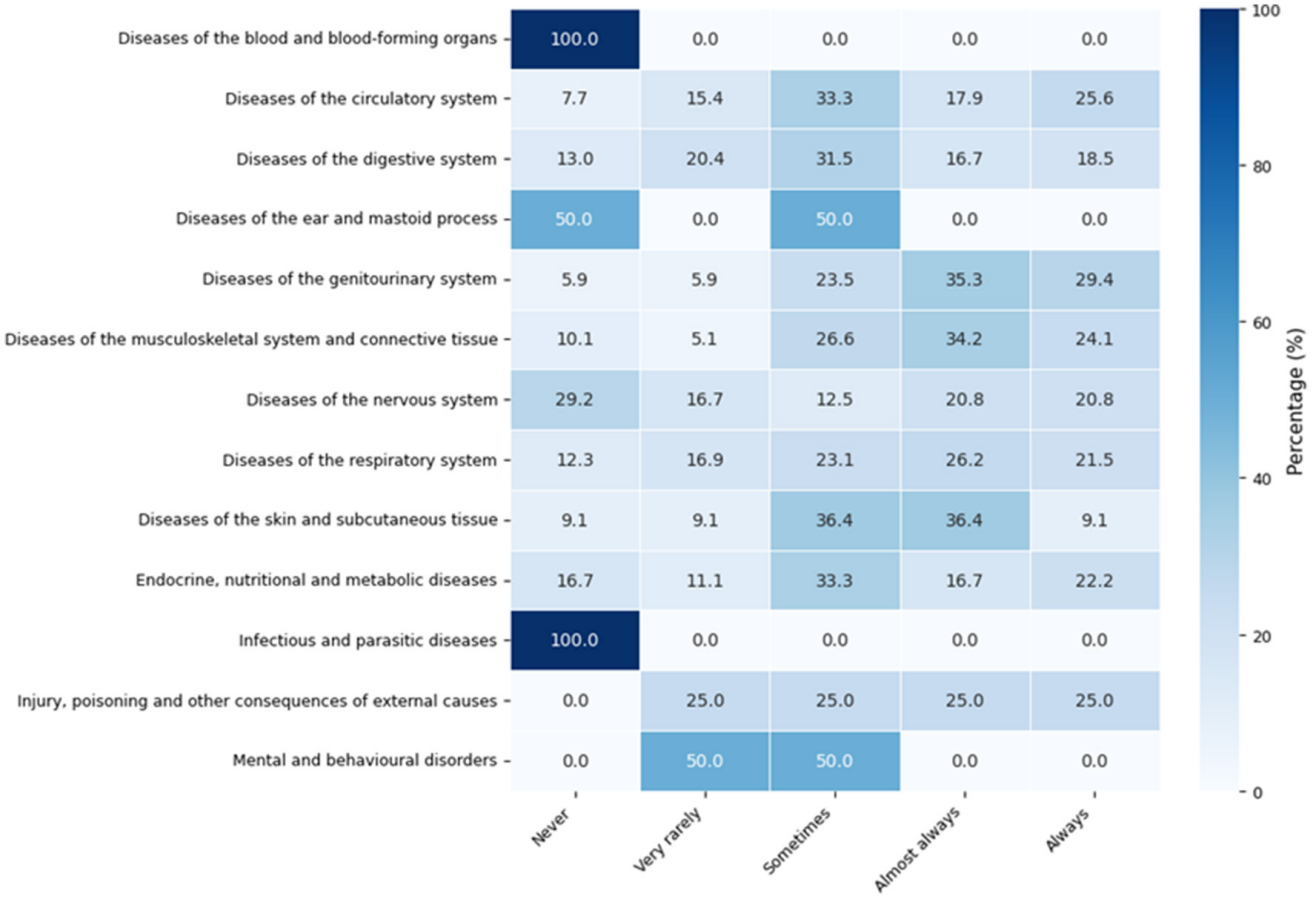
Percentage distribution of medicinal plant use frequency according to disease categories classified by the International Classification of Diseases (ICD-10). Values represent percentages within each disease category.

Figure 8 presents the percentage distribution of medicinal plant use frequency across disease categories classified according to ICD-10 chapters. The percentages represent the relative distribution of use within each disease category, allowing for the comparison of usage patterns across different groups of reported conditions. The results show variations in medicinal plant use according to disease type. Higher proportions of frequent use (“sometimes,” “almost always,” or “always”) are observed in diseases of the digestive, respiratory, and genitourinary systems. In contrast, other categories tend to concentrate in lower levels of reported use, reflecting differences in perceived need or customary practices associated with each type of condition. These findings highlight differentiated patterns of medicinal plant use across disease categories, providing insight into how participants adapt their use of traditional remedies depending on the type of health condition reported.

**Figure 9:**
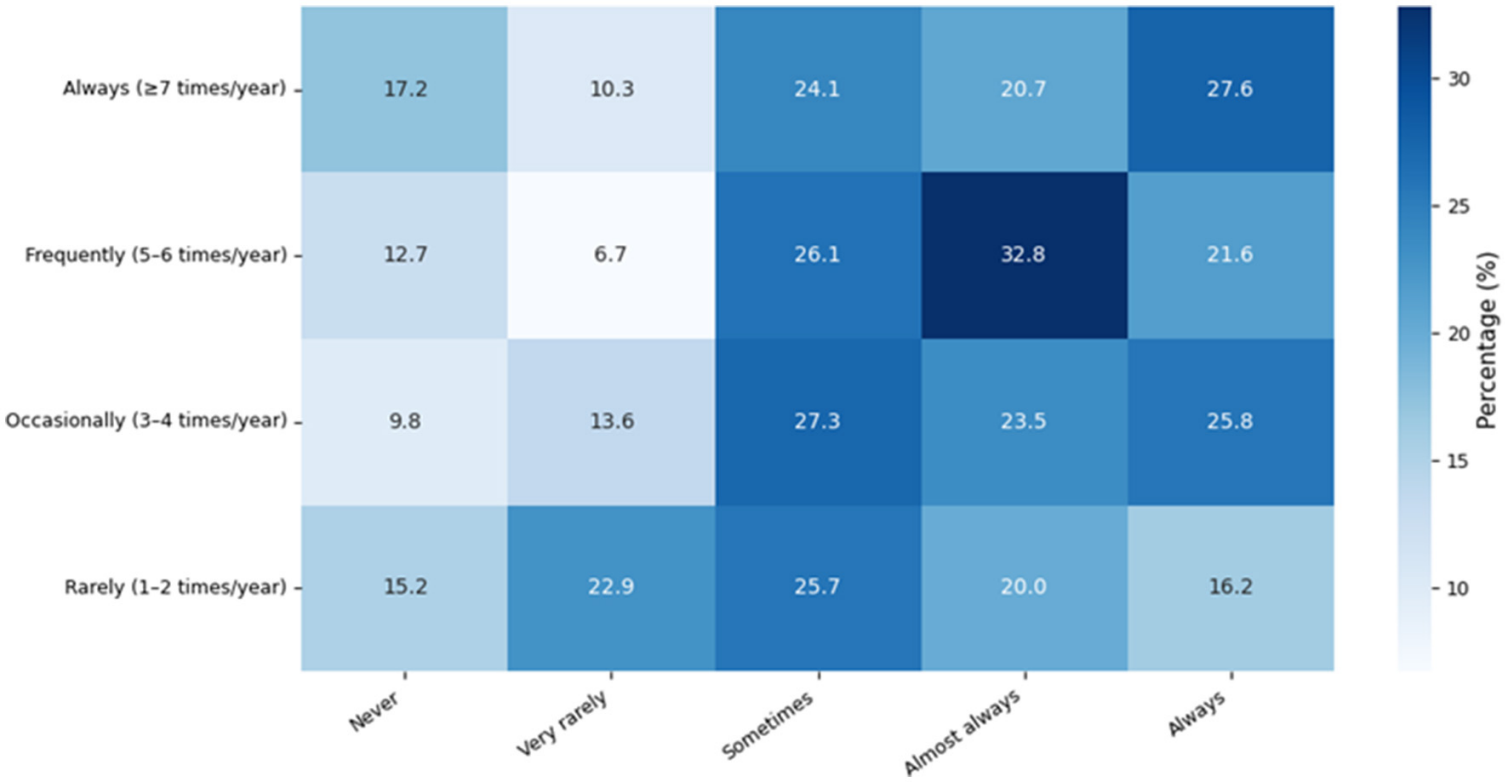
Association between disease frequency and medicinal plant use frequency in the study population. Values are percentages per disease frequency category.

Figure 9 presents the distribution of medicinal plant use frequency according to the recurrence of illness episodes reported by participants. Differences in the distribution of use are observed across levels of illness recurrence. Higher recurrence categories show greater proportions in intermediate and high levels of medicinal plant use, while lower recurrence categories tend to concentrate relatively in lower levels of use. These patterns illustrate variations in the frequency of medicinal plant use across groups defined by illness recurrence.

**Figure 10:**
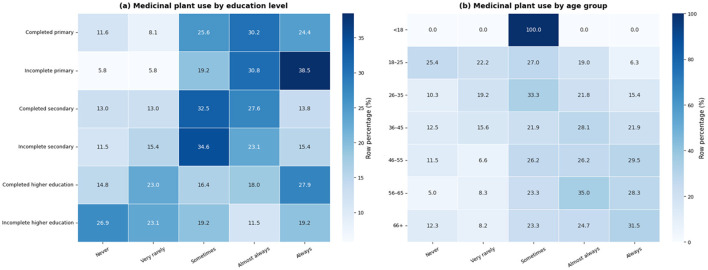
Distribution of the frequency of medicinal plant use according to educational level and age group. **(a)** Distribution of the frequency of medicinal plant use according to educational level. **(b)** Distribution of the frequency of medicinal plant use according to age group. Values are percentages per row.

Figure 10 presents the distribution of medicinal plant use frequency according to participants' educational level and age group. Differences in the distribution of use are observed across educational levels, with variations in the proportion of responses across the different categories of use. Similarly, variations in the distribution of medicinal plant use are observed across age groups, with differences in how responses are distributed among the categories of use. These patterns reflect heterogeneity in the distribution of medicinal plant use across sociodemographic groups within the study sample.

**Figure 11:**
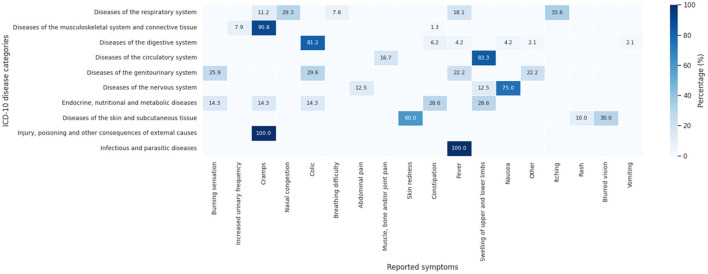
Distribution of reported symptoms according to the International Classification of Diseases (ICD-10) disease categories.

Figure 11 reports the distribution of symptoms declared by participants according to disease categories classified using ICD-10 chapters. In diseases of the respiratory system, nasal congestion (29.3%), itching (33.6%), and fever (18.1%) show higher proportions, along with cramps (11.2%) and breathing difficulty (7.8%). In diseases of the musculoskeletal system and connective tissue, cramps account for 90.8% of reported symptoms, while in diseases of the digestive system, colic represents 81.2%, followed by lower proportions of constipation (6.2%), nausea (4.2%), and vomiting (2.1%).

Diseases of the circulatory system show higher proportions of swelling of upper and lower limbs (83.3%) and muscle, bone and/or joint pain (16.7%). In diseases of the genitourinary system, burning sensation (25.9%) and colic (29.6%) are among the most frequently reported symptoms, while diseases of the nervous system show a higher proportion of nausea (75.0%). Endocrine, nutritional and metabolic diseases show similar proportions of constipation (28.6%) and nausea (28.6%), whereas diseases of the skin and subcutaneous tissue are associated with skin redness (60.0%). Infectious and parasitic diseases show a higher proportion of fever (100%). These results present the distribution of reported symptoms across disease categories within the study sample.

The chi-square test of association was conducted to examine the relationship between medicinal plant consumption and several sociodemographic and health-related variables. The results of these analyses are presented in Table 2. Statistically significant associations were identified between medicinal plant consumption and sex (χ^2^ = 14.429; *p* = 0.006), educational level (χ^2^ = 40.923; *p* = 0.004), illness duration (χ^2^ = 22.609; *p* = 0.004), reported symptoms (χ^2^ = 45.782; *p* < 0.001), frequency of visits to health centers (χ^2^ = 32.575; *p* < 0.001), perceived usefulness of medicinal plants (χ^2^ = 474.070; *p* < 0.001), and forms of medicinal plant consumption (χ^2^ = 336.695; *p* < 0.001).

**Table 2:** Chi-square tests of association between medicinal plant use and sociodemographic and health-related variables.

Variable	χ^2^	df	p-value	Cramér's V
Sociodemographic variables				
Sex	14.429	4	0.006^*^	0.190
Neighborhood	17.268	12	0.140	0.120
Educational level	40.923	20	0.004^*^	0.160
Health-related variables				
Disease frequency	20.972	12	0.051	0.132
Illness duration	22.609	8	0.004^*^	0.168
Impact on daily activities	9.013	4	0.061	0.150
Days of absence	15.585	12	0.211	0.114
Visits to health center (grouped)	32.575	8	< 0.001^*^	0.202
Symptoms (grouped)	45.782	20	< 0.001^*^	0.195
Diseases (grouped)	19.126	20	0.514	0.109
Medicinal plant use variables				
Perceived usefulness of medicinal plants	474.070	16	< 0.001^*^	0.544
Form of medicinal plant consumption (grouped)	336.695	8	< 0.001^*^	0.649

^*^Statistically significant association (*p* < 0.05).

Some variables were grouped to reduce sparse cell counts and satisfy the chi-square assumption of ≤ 20% expected frequencies below 5.

For variables with a large number of categories (e.g., symptoms, diseases, forms of consumption, and visits to health centers), categories were grouped prior to analysis to satisfy the chi-square assumption regarding expected cell frequencies. This grouping was performed using predefined criteria based on conceptual similarity and distributional balance, ensuring that no more than 20% of expected cell frequencies were below 5.

Specifically, variables such as ICD-10 classification and forms of consumption were collapsed into broader categories to reduce sparsity and improve the robustness of the analysis. The grouping process was applied systematically and consistently across variables. The magnitude of the associations, estimated using Cramér's V, ranged from small to large effect sizes. Given the number of comparisons performed, results were interpreted as indicative of general association patterns rather than isolated statistical significance. It should be noted that variables such as perceived usefulness and form of consumption are conceptually related to medicinal plant use, and therefore their associations should be interpreted within this contextual relationship.

Table 3 shows the results of the ordinal logistic regression model estimated using maximum likelihood (Log-Likelihood = −604.48; AIC = 1,227; BIC = 1,263). The findings indicate that age and sex are significantly associated with the level of medicinal plant use. Specifically, age exhibits a positive and statistically significant effect (β = 0.0219; *p* < 0.001), indicating that increasing age is associated with a higher probability of being in higher consumption categories. In contrast, sex shows a significant negative coefficient (β = −0.5978; *p* = 0.001), suggesting differences in the distribution of use across this variable.

**Table 3:** Results of the ordinal logistic regression model for medicinal plant use.

Model information						
Dependent variable	Medicinal plant use		Log-Likelihood		−604.48	
Model	Ordinal logistic regression		AIC		1,227	
Method	Maximum likelihood		BIC		1,263	
No. Observations:	400					
Df Residuals:	391					
Df Model:	5					
Variable	Coefficient (β)	Standard error	*z*	*P* > |*z*|	[0.025	0.975]
Sex	−0.5978	0.184	−3.249	0.001^*^	−0.959	−0.237
Age	0.0219	0.006	3.753	0.000^*^	0.010	0.033
Education level	−0.0531	0.066	−0.801	0.423	−0.183	0.077
Health center visits	0.0194	0.119	0.163	0.87	−0.214	0.253
Illness period	0.217	0.132	1.64	0.101	−0.042	0.476
1/2	−1.6267	0.541	−3.007	0.003	−2.687	−0.567
2/3	−0.0663	0.130	−0.510	0.610	−0.321	0.188
3/4	0.2162	0.089	2.436	0.015	0.042	0.390
4/5	0.2360	0.090	2.609	0.009	0.059	0.413

^*^p < 0.05. The dependent variable was coded as a five-level ordinal scale (never, very rarely, sometimes, almost always, always). In this context, the parameters labeled 1/2, 2/3, 3/4, and 4/5 correspond to the cut points of the model, defining the transitions between adjacent ordinal categories. These thresholds are part of the ordinal logistic model structure and do not represent direct effects of the explanatory variables.

Education level, health center visits, and illness period do not show statistically significant associations (*p* > 0.05), indicating a limited contribution to explaining variation in medicinal plant use within the model.

Some threshold parameters are statistically significant, supporting the differentiation between the ordinal categories of the dependent variable. Overall, the results reflect variation in medicinal plant use across sociodemographic characteristics, while no consistent patterns are observed for health-related variables. The selection of variables included in the multivariable model was guided by their analytical relevance and the structure of the data, ensuring model parsimony and coherence in interpretation. In this context, priority was given to sociodemographic and health-related variables to assess associations within the analytical framework of the study.

Table 4 shows the odds ratios derived from the ordinal logistic regression model. The results confirm that age is positively associated with medicinal plant use (OR = 1.022; 95% CI: 1.011–1.034; *p* < 0.001), indicating that increasing age is associated with a higher probability of being in higher consumption categories. In contrast, sex presents an odds ratio below one (OR = 0.550; 95% CI: 0.383–0.789; *p* = 0.001), reflecting differences in the probability of use across this variable.

**Table 4:** Odds ratios of the ordinal logistic regression model for medicinal plant use.

Variable	Coeficiente	OR	IC_2.5%	IC_97.5%	*p*_value
Sex	−0.598	0.550	0.383	0.789	0.001^*^
Age	0.022	1.022	1.011	1.034	0.000^*^
Education level	−0.053	0.948	0.833	1.080	0.423
Health center visits	0.019	1.020	0.808	1.287	0.870
Illness period	0.217	1.242	0.959	1.610	0.101
1/2	−1.627	0.197	0.068	0.568	0.003^*^
2/3	−0.066	0.936	0.726	1.207	0.610
3/4	0.216	1.241	1.043	1.477	0.015^*^
4/5	0.236	1.266	1.060	1.512	0.009^*^

^*^p < 0.05.

Education level, health center visits, and illness period do not show statistically significant associations (*p* > 0.05), as their confidence intervals include the null value (OR = 1), indicating no clear effect within the model. The threshold parameters define the cut points between ordinal categories of medicinal plant use and are intrinsic to the model specification rather than representing direct effects of the explanatory variables.

## 4 Discussion

Regarding the sociodemographic characteristics of the population, a higher proportion of women and a predominance of older adults were observed, with education levels primarily corresponding to secondary schooling. Although this distribution is consistent with ethnobotanical literature highlighting the central role of women in the transmission of traditional knowledge related to medicinal plants (61–65). At the same time, these findings should be interpreted considering the study context, as participation was based on household visits and voluntary response. Under these conditions, the observed distribution may reflect both underlying sociocultural patterns and participant availability during data collection. Therefore, the results remain consistent with the literature, while also acknowledging the influence of contextual factors inherent to field-based data collection in rural settings.

In this context, the findings highlight the relevance of women's roles in preserving and transmitting traditional medicine practices in rural communities. This observation opens opportunities to further explore the socioeconomic roles of traditional medicine for women across the coastal, Andean, and Amazonian regions of Peru, as well as the structural barriers they face in integrating their practices within formal healthcare systems. Understanding these dynamics could contribute to the development of more inclusive and culturally appropriate health systems oriented toward sustainable development.

The observed patterns of illness frequency, healthcare utilization, and duration of illness episodes suggest a dynamic interaction between morbidity burden and health-seeking behavior in rural contexts, where recurrent but generally mild conditions, particularly respiratory and gastrointestinal, generate sustained demand for care. These findings are consistent with evidence indicating that such morbidity patterns in rural populations are influenced by environmental exposure and limited continuity in healthcare access (66–68). However, rather than reflecting purely clinical needs, these patterns must be interpreted within a broader sociocultural and structural framework, where individuals combine biomedical services with self-care strategies such as medicinal plant use (5, 9). In this context, the absence of statistically significant associations between illness frequency and medicinal plant use suggests that these practices are not solely reactive to disease recurrence, but are embedded in culturally shaped behaviors and perceptions of effectiveness, consistent with studies highlighting the influence of accessibility, cultural acceptability, and experiential knowledge in traditional medicine use (6, 7).

Likewise, several studies have indicated that educational level is associated with differences in the use of traditional health practices, particularly in contexts where access to specialized medical services is limited (7, 62, 69). In this regard, the results obtained suggest the presence of variation in the patterns of use of traditional health practices across educational groups. However, this association should be interpreted within a broader health-behavior framework, as educational level may reflect underlying differences in access to health information, cultural norms, and contextual conditions related to healthcare availability, rather than acting as a direct determinant of behavior.

With respect to the frequency of illnesses and the utilization of healthcare services, the findings reveal the presence of occasional or recurrent episodes of illness during the analyzed period, accompanied by relatively frequent visits to health centers and episodes that were generally short in duration (70). This pattern is consistent with studies conducted in rural populations, which indicate that the presence of mild but recurrent morbidity is often associated with specific social, occupational, and environmental conditions, as well as with limitations in continuous access to healthcare services (66–68). In this context, the coexistence of reported use of health services and medicinal plants suggests the presence of diversified health-seeking strategies commonly described in rural settings. However, given the design of the study, it is not possible to determine the sequencing, substitution, or concurrent use of these practices. Therefore, the findings should be interpreted as indicative of parallel use patterns rather than as direct evidence of functional complementarity between biomedical and traditional care. This situation highlights the need to develop more detailed approaches to better understand the role of indigenous traditional medicine within contemporary healthcare systems.

Regarding the classification of diseases according to ICD-10, and reported symptoms, respiratory, gastrointestinal, and chronic non-communicable diseases predominate, often accompanied by symptoms such as fever, nasal congestion, abdominal cramps, and pain. This clinical profile is similar to recent epidemiological research conducted in rural areas of Peru and other Andean nations, where digestive and respiratory infections remain common reasons for seeking medical consultation (26, 71) alongside chronic diseases associated with population aging and changes in lifestyle habits (72). Although several studies have explored the pharmacological potential of natural products used in traditional medicine, it is important to note that such evidence is typically species-specific and context-dependent. In the study, the use of medicinal plants was assessed at a general level based on participant self-report, without species identification or pharmacological validation; therefore, the findings do not allow for inferring bioactivity, safety, or therapeutic efficacy of specific plant species. Recent clinical and translational literature emphasizes the need for direct evidence in herb-related therapeutic decisions, rather than broad extrapolation from traditional use alone (73, 74).

Traditional health practices continue to play a relevant role within the sociocultural context of rural communities. In this sense, the use of medicinal plants can be interpreted as an accessible strategy for symptom management. However, the study design does not allow for determining whether these practices are used as first-line treatment, as a complementary option, or as an alternative when biomedical care is not accessible. These findings reinforce the importance of continuing to investigate traditional health practices using designs that capture treatment pathways and decision-making processes, in order to better understand their role within local healthcare systems and their potential contribution to health education and community well-being.

The functional impact of the reported illnesses suggests that even short-duration episodes may significantly affect daily activities, as well as attendance at work or school. Previous studies have indicated that, in populations whose livelihoods depend largely on daily rural labor, even minor illnesses may have meaningful consequences for productivity and quality of life (75). In this context, the search for accessible and immediate remedies, such as medicinal plants, may constitute an adaptive response to structural limitations in access to healthcare services.

Regarding the use of medicinal plants, a high frequency of consumption was identified, accompanied by a largely positive perception of their usefulness. Traditional preparation methods predominated, particularly infusions and decoctions. These results reflect patterns of use reported by participants within their sociocultural context, highlighting the relevance of traditional preparation methods such as infusions and decoctions in everyday health practices. Studies conducted in diverse cultural contexts, including Pakistan, Algeria, Saudi Arabia, Lithuania, South Africa, and Iran, have documented that infusion and decoction are among the most common preparation methods for herbal remedies (38, 76–79). The predominance of these preparation methods reflects the persistence of culturally transmitted practices across generations, in which phytotherapy constitutes a widely accepted therapeutic resource within the community. From a translational perspective, however, the high prevalence of use and perceived usefulness should not be interpreted as evidence of therapeutic efficacy, but rather as indicative of culturally embedded health behaviors that may generate hypotheses for future research.

Regarding the type of illnesses, respiratory, digestive, and genitourinary disorders were most frequently associated with the use of medicinal plants. This pattern is consistent with recent ethnopharmacological research documenting the widespread use of medicinal plants to treat such conditions (73, 80). While many plant species have been reported to contain bioactive compounds with antimicrobial (81), antispasmodic, expectorant, and anti-inflammatory properties (82–84), these properties are species-specific and context-dependent and were not directly assessed in this study. In this sense, rather than inferring clinical effectiveness, the observed patterns may be interpreted as consistent with potential biological pathways, such as modulation of inflammatory responses, protection of epithelial barriers, and microbiota-mediated effects, that have been highlighted in recent translational research on plant-based interventions (85). Furthermore, medicinal plant use should not be understood as an isolated exposure, but as part of complex interactions with host physiological and metabolic states, particularly in the context of chronic disease vulnerability and symptom patterns (86). These considerations support the need for future studies integrating ethnobotanical data with experimental and clinical evidence to better understand the mechanisms underlying the reported use of medicinal plants.

Additionally, variations in the frequency of medicinal plant use were observed according to the recurrence of illness episodes. Individuals experiencing more frequent illnesses tended to rely more regularly on these therapeutic resources. This behavior has been documented in recent studies, where the recurrence of illness acts as a factor promoting the use of complementary healthcare practices, particularly when conventional treatments do not completely resolve symptoms or involve additional financial costs.

In line with these findings, the chi-square analysis further highlights that medicinal plant use is associated with multiple sociodemographic and health-related factors, including sex, educational level, illness duration, and healthcare utilization. This supports previous research indicating that traditional medicine practices are shaped by a complex interaction of cultural norms, access to healthcare, and individual perceptions of health (4, 5). In rural settings characterized by structural and geographic barriers, these factors become particularly relevant in shaping health-seeking behavior (54). At the same time, the lack of significant associations with disease type and frequency reinforces the idea that medicinal plant use is not exclusively determined by clinical need, but rather by culturally embedded practices and accumulated experiential knowledge (6, 7).

Differences observed in medicinal plant use according to age and educational level reflect not only sociocultural patterns but also functional dimensions associated with aging. While traditional knowledge about medicinal plants is often concentrated among older generations (87), and among groups with lower educational attainment, which is consistent with studies conducted in various rural contexts examining the traditional use of medicinal plants (62, 88), this pattern may also be influenced by age-related factors such as greater chronic symptom burden, reduced mobility, differences in daily activity patterns, and increased reliance on local social networks for health-related decision-making. In this sense, the higher use observed among older adults should not be interpreted solely as a reflection of cultural knowledge, but rather as the result of a combination of experiential, functional, and contextual factors. Recent perspectives emphasize the importance of considering time-use patterns and active-life engagement when analyzing health behaviors in aging populations (89), which may help to better contextualize the role of medicinal plants within everyday health practices.

This interpretation is further supported by the ordinal logistic regression results, which identify age and sex as the main predictors of medicinal plant use when controlling for other variables. The positive effect of age reflects the accumulation and transmission of traditional knowledge among older populations (46, 87), while the effect of sex is consistent with differentiated roles in household health decision-making and care practices (45, 56). In contrast, variables such as education, healthcare visits, and illness duration lose statistical significance in the multivariable model, suggesting that their influence may be mediated by broader sociocultural factors. This aligns with previous studies indicating that traditional medicine use responds to a complex interplay of demographic, cultural, and accessibility-related determinants rather than isolated factors (9, 11).

These findings highlight intergenerational and educational differences in the preservation of traditional knowledge, an important consideration for public health research. Rapid urbanization and formal schooling may concentrate this knowledge among older populations, underscoring the need to continue documenting traditional health practices across sociocultural contexts. The patterns observed in Figure 9 align with global evidence showing that culture, age, and education shape health care practices, with medicinal plant use maintaining strong social significance. These results also emphasize the need to move beyond descriptive approaches toward integrative frameworks that incorporate environmental exposure, mobility, and community structure. In rural settings, health-seeking behavior is influenced not only by disease occurrence but also by access networks, mobility constraints, and local context. Although these factors were not measured, their inclusion in future research would strengthen understanding of health practices in complex socio-environmental systems. Additionally, computational prioritization approaches may help identify plant–condition relationships and generate testable hypotheses, supporting the transition toward more analytical and predictive research frameworks (90).

From a broader analytical perspective, the findings also highlight the need to move beyond descriptive prevalence approaches toward more integrative models that incorporate environmental exposure, mobility patterns, and community structure in the analysis of health-seeking behavior. In rural contexts, treatment decisions are not determined solely by disease occurrence but are also shaped by network-based access to care, geographic and mobility constraints, and neighborhood-level dynamics. These structural and contextual dimensions were not directly measured in the present study, representing an important avenue for future research. Incorporating such factors into analytical frameworks would allow for a more comprehensive understanding of how health behaviors are configured in rural settings and would strengthen the interpretation of medicinal plant use within complex socio-environmental systems (91, 92).

Although this study has several limitations, these should be interpreted within the context of its analytical approach. The cross-sectional design precludes causal inference; however, the use of chi-square tests and multivariable ordinal logistic regression allowed the identification of statistically significant associations while controlling for potential confounders. Data were based on self-reported information, which may introduce recall or reporting bias, and the use of non-probability sampling restricted to individuals with at least one reported illness limits generalizability to the broader population. In addition, illness episodes may be subject to overlapping or repeated reporting, and disease classification, although aligned with ICD-10, was based on reported symptoms rather than clinical diagnosis. The study did not include plant species identification or pharmacological validation, preventing conclusions regarding safety or efficacy. Despite these constraints, the use of standardized instruments and multivariable analytical methods provides robust evidence on patterns of medicinal plant use and their associations with reported morbidity in a rural context.

## 5 Conclusions

The morbidity profile of the rural community studied is characterized predominantly by respiratory, digestive, and genitourinary conditions, which are generally of short duration but have a measurable impact on daily activities and productivity. These patterns reflect the persistence of common, recurrent health conditions that shape health-seeking behavior in rural contexts.

Medicinal plant use was identified as a widespread and socially embedded practice, with a predominance of traditional preparation methods such as infusions and decoctions, highlighting the continuity of culturally transmitted self-care practices. The frequency of use varies across disease categories and recurrence patterns, suggesting its role as part of locally reported symptom management behaviors rather than indicating condition-specific therapeutic effectiveness.

The bivariate analysis (chi-square) identified significant associations between medicinal plant use and several sociodemographic and health-related variables, including sex, educational level, illness duration, healthcare utilization, and perceived usefulness. Higher use was observed in respiratory, digestive, and genitourinary conditions, particularly among individuals reporting recurrent illness episodes.

The multivariable ordinal logistic regression model identified age and sex as the only significant predictors after adjustment for confounders. Other variables lost statistical significance, indicating that their effects are explained by broader sociocultural and contextual dynamics. Medicinal plant use is structured primarily by demographic and social factors rather than by direct indicators of disease burden.

The study's findings highlight the sociocultural importance of medicinal plant use as part of self-care practices in rural communities. In this context, these resources constitute a relevant component of local strategies for symptom management and health care within the traditional practices present in the community.

The results provide empirical evidence on the patterns of medicinal plant use in relation to the illnesses reported by the population, contributing to a better understanding of local self-care practices in rural contexts. Although the study did not include pharmacological or clinical evaluations of the species used, the findings offer relevant information to guide future research aimed at characterizing their therapeutic efficacy, safety, and potential integration into evidence-based health strategies.

## Data Availability

The original contributions presented in the study are included in the article/supplementary material, further inquiries can be directed to the corresponding author.
